# New antibiotics in clinical pipeline for treating infections caused by metallo-β-lactamases producing Gram-negative bacteria

**DOI:** 10.1097/QCO.0000000000001056

**Published:** 2024-09-12

**Authors:** Matteo Bassetti, Antonio Vena, Barbara Larosa, Daniele Roberto Giacobbe

**Affiliations:** a Department of Health Sciences (DISSAL), University of Genoa; b Clinica Malattie Infettive, IRCCS Ospedale Policlinico San Martino, Genoa, Italy

**Keywords:** antimicrobial resistance, aztreonam/avibactam, aztreonam/nacubactam, carbapenemases, cefepime/nacubactam, cefepime/taniborbactam, cefepime/zidebactam, cefiderocol, novel agents

## Abstract

**Purpose of review:**

To discuss novel antibiotics under clinical development, focusing on agents showing in-vitro activity against metallo-β-lactamases (MBL)-producing carbapenem-resistant Gram-negative bacteria (CR-GNB).

**Recent findings:**

Currently, only a few approved agents show activity, alone or in synergistic combinations, against MBL-producing CR-GNB. If approved by regulatory agencies in case of favorable results from ongoing (and, for some agents, already completed) phase-3 studies, some novel β-lactam/β-lactamase inhibitor (BL/BLI) combinations could become available in the next few years as additional important options for treating MBL-producing CR-GNB infections. Additional interesting agents that belong both to BL/BLI combinations and to antibiotic classes other than BL and BL/BLI combinations have also shown activity against MBL-producing CR-GNB, with most of them being in early phases of clinical development.

**Summary:**

Improving the use of these novel agents through virtuous antimicrobial stewardship frameworks able to guarantee both the efficacious treatment of infections requiring their use and the avoidance of their use whenever not necessary remains a challenge of utmost importance that should not be overlooked.

## INTRODUCTION

Starting from the last few years, the first-line treatment of infections caused by carbapenem-resistant Gram-negative bacteria (CR-GNB) has witnessed an increasingly consistent shift from polymyxin-based regimens to novel β lactam based agents, in line with their reduced nephrotoxicity in comparison with the former, and with the most recent guidelines and guidance documents from international scientific societies [1–3].

The possibility of treating CR-GNB infections again with β-lactams (BL) or BL/β-lactamase inhibitor (BL/BLI) combinations has also changed how clinicians conceptually face the clinical reasoning about which agent/s to employ. Indeed, crucial importance is now conferred to the type of expressed carbapenemases when carbapenem resistance is due to these enzymes [4,5]. This inherently follows the differential activity of the various novel BL and BL/BLI combinations based on the type/s of expressed carbapenemases. Against this background, only a few marketed agents show activity, alone or in synergistic combinations, against metallo-β-lactamases (MBL)-producing CR-GNB [6]. Consequently, there is great interest in the development of additional novel agents active against these organisms, to further expand our armamentarium and curtail once again the risk of emerging pan-resistance in the long-term.

In this brief narrative review, we discuss novel antibiotics under clinical development, focusing on agents showing in-vitro activity against MBL-producing CR-GNB. 

**Box 1:**
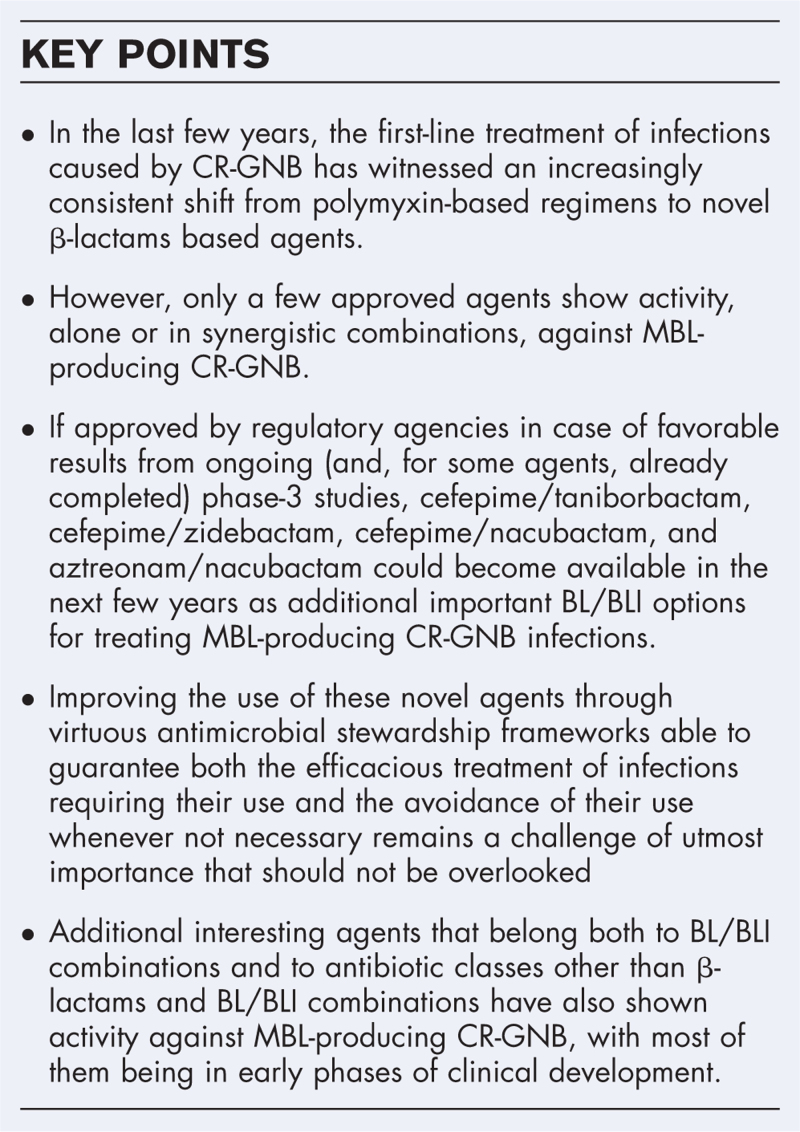
no caption available

## AGENTS UNDER CLINICAL DEVELOPMENT WITH AT LEAST ONE CONCLUDED PHASE-3 TRIAL

Cefepime/taniborbactam is a novel BL/BLI combination including taniborbactam, a novel BLI able to inhibit also MBLs, with the notable exception of IMP-type enzyme, in Enterobacterales members and in *Pseudomonas aeruginosa*[7,8]. Cefepime/taniborbactam is under evaluation by the Food and Drug Administration (FDA) regarding marketing authorization for the treatment of complicated urinary tract infections (cUTI) and acute pyelonephritis (AP) in adult patients [9]. The request for marketing authorization is based on the results of the phase-3, double-blind, randomized controlled trial (RCT) CERTAIN-1 in which cefepime/taniborbactam was noninferior (and also superior based on a predefined analysis in case of achievement of noninferiority) to meropenem for the treatment of cUTI/AP [10^▪^]. Notably, carbapenem-resistant infections were excluded from the primary study population (microbiological intent-to-treat [microITT], which included cUTI/AP patients with infection by organisms susceptible both to cefepime/taniborbactam and to meropenem). An extended mITT population included also carbapenem-resistant infections treated with cefepime/taniborbactam, which nonetheless were only 10 (mostly caused by *Klebsiella pneumoniae*) and without specific determinants of carbapenem resistance being reported. Composite success (clinical and microbiological) at test of cure was registered in eight of 10 patients with carbapenem-resistant infections treated with cefepime/taniborbactam (80%). In a second phase-3 RCT (CERTAIN-2), which is expected to start within the end of 2024, the efficacy of cefepime/taniborbactam vs. meropenem will be assessed in patients with hospital-acquired pneumonia (HAP), including ventilator-associated pneumonia (VAP) [11].

Avibactam is a nonβ-lactam BLI able to inhibit class A, C, and some class D enzymes, but not MBLs [12]. However, its combination with the well known monobactam aztreonam shows activity against MBL-producing CR-GNB [12,13]. This is due to the inability of MBLs to hydrolyze aztreonam, which is then protected by avibactam from inactivation by various serine-β-lactamases, that can conversely hydrolyze aztreonam and are frequently co-expressed by MBL-producing CR-GNB [12]. The European Medicines Agency (EMA) has recently approved aztreonam/avibactam for the treatment of cUTI/AP, complicated intraabdominal infections (cIAI), HAP, VAP, and infections by aerobic GNB with limited treatment options in adults [14]. The approval is based on the results of two phase-3 studies, REVISIT and ASSEMBLE. In the open-label RCT REVISIT, aztreonam/avibactam (and metronidazole in the case of cIAI) was compared to meropenem (± colistin) in the treatment of infections (cIAI, HAP, VAP) by GNB in adults, showing similar adjusted clinical cure rates (primary efficacy endpoint) across study arms (68.4% [193/282] and 65.7% [92/140] in interventional and comparator arms, respectively) in the primary study population (intent-to-treat) [15^▪^,^16^]. No formal evaluation of superiority or noninferiority was planned. Only five patients had infections caused by MBL-producing organisms, four in the aztreonam/avibactam arm (2/4 achieved clinical cure, 50%) and one in the meropenem ± colistin arm (0/1 achieved clinical cure, 0%) [15^▪^,^16^]. The open-label ASSEMBLE RCT, aimed at comparing aztreonam/avibactam with best-available therapy for the treatment of infections by MBL-producing organisms, was early terminated due to enrollment challenges (with the relevant limitation of the small sample size, 5/12 patients [42%] achieved clinical cure in the aztreonam/avibactam arm vs. 0/3 [0%] in the best available therapy arm) [17].

## AGENTS IN PHASE-3 OF CLINICAL DEVELOPMENT

Aztreonam is also under phase-3 of clinical development combined with nacubactam, a BLI with no inhibitory activity against MBLs, but able to protect aztreonam from hydrolysis by serine β-lactamases [18–20]. It is nonetheless of note that also the combination of nacubactam with cefepime (which has also entered phase-3 of clinical development) has shown activity *in vitro* against MBL-producing members of the Enterobacterales [21,22]. This peculiar activity (unexpected, since cefepime is unable to resist hydrolysis by MBLs) seems to be conferred by a β-lactams activity of nacubactam, mediated by its affinity for penicillin-binding protein 2 (PBP2) and enhancing effect. Of note, contrarily to what observed for Enterobacterales, not improved activity over cefepime has been observed for cefepime/nacubactam against *P. aeruginosa*[21,22].

Cefepime has also showed activity against MBL-producing CR-GNB (both against members of the Enterobacterales and against *P. aeruginosa*) when combined with the diazabicyclooctane derivative zidebactam, another novel BLI showing beta-lactam activity through affinity for PBP2 [23,24]. Although the combination is still in phase-3 of clinical development [25], a few real-life reports showing clinical success after compassionate use of cefepime/zidebactam as salvage therapy for MBL-producing *P. aeruginosa* infections are available in the literature [26–28].

## AGENTS IN EARLIER PHASES OF CLINICAL DEVELOPMENT

Before being assessed in combination with aztreonam or cefepime, nacubactam had already been investigated in combination with meropenem, although no phase-3 RCTs are currently available and the combination is currently reported to be in phase-1 of clinical development [29,30]. Of note, enhancement of β-lactam activity by nacubactam has been observed in combination with aztreonam, piperacillin, and cefepime, but not with meropenem (the underlying hypothesis of such a differential enhancement is that of a dual β-lactam action of nacubactam binding PBP2 and aztreonam, piperacillin, or cefepime binding PBP3 primarily, while meropenem shows high affinity for PBP2) [20,21].

Meropenem has entered phase-1 of clinical development also in combination with a novel BLI (KSP-1007), with the combination showing wide spectrum inhibition of serine-β-lactamases and MBLs in in-vitro studies [30,31].

Apart from being developed in combination with cefepime, the BLI zidebactam is also under clinical development in combination with ertapenem (phase-1), with in-vitro studies reporting activity against MBL-producing Enterobacterales [32,33].

Xeruborbactam is a bicyclic boronate BLI which is under clinical development (phase-1) in combination with cefiderocol (intravenously) and with ceftibuten (orally) [30,34–39]. Xeruborbactam is able to inhibit various β-lactam, including MBLs such as NDM and VIM [34–39].

Activity against MBL producers is also expected from some novel agents undergoing clinical development that belong to antibiotic classes other than β-lactams and BL/BLI combinations. Indeed, since they do not exhibit a β-lactams structure, the presence of MBLs is not a factor conferring resistance *per se*, and activity is dependent of the lack or avoidance of other resistant mechanisms (different from β-lactamases) able to confer resistance to other antibiotic classes. For example, although hampered by a nonnegligible risk of nephrotoxicity, possible neurotoxicity, and possible suboptimal efficacy for various reasons, polymyxins (which disrupt the outer cell membrane of GNB thereby increasing permeability) were frequently used in the past for treating infections by MBL-producing CR-GNB (when they were among the very few dependable options and the recently marketed β-lactams and BL/BLI combinations were still unavailable) [40–43]. With regard to polymyxin derivatives under clinical development, upleganan, MRX-8, and QPX9003 are in phase-1 (completed), phase-1 (completed), and phase-1 of clinical development, respectively, with some preliminary evidence suggesting possible reduced toxicity in comparison with currently available polymyxins [30,44–46].

In-vitro activity against CR-GNB has also been preliminary showed by apramycin (EBL-1003), an aminoglycoside in phase-1 of clinical development [47,48].

The third-generation aminomethylcycline zifanocycline (KBP-7072) is currently in phase-1 of clinical development, and has shown in-vitro activity against MBL-producing *Acinetobacter baumannii*[30,49,50]. Zifanocycline seems to be affected only minimally by acquired tetracycline resistance genes [51].

BWC0977 is a novel nonfluoroquinolone topoisomerase inhibitor with reported activity against various GNB that has entered phase-1 of clinical development [30]. Nonetheless, no peer-reviewed publications and information about any possible activity against MBL-producing CR-GNB are currently available.

Regarding agents with novel mechanisms of action, RECCE 327 is a synthetic polymer that disrupt bacterial energy production, cell growth, and cell division, reported to show in-vitro activity against CR-GNB, although no peer-reviewed publications and information about any possible activity against MBL-producing CR-GNB are currently available [30]. OMN6, a synthetic cyclic peptide showing antibacterial activity by disrupting bacterial membranes, exhibits in-vitro activity against various GNB, and is currently in phase-1 of clinical development [52,53]. Finally, murepavadin, a peptidomimetic agent that inhibits an outer membrane protein in GNB (LptD, involved in the synthesis of lipopolysaccharide in *P. aeruginosa*), was previously halted in its development as an intravenous formulation owing to safety issues, and is currently in phase 1b/2a of clinical development as an inhaled formulation [30,54–57].

## CURRENT SCENARIO AND THE POTENTIAL PLACE IN THERAPY OF FUTURE AGENTS FOR THE TREATMENT OF METALLO β LACTAMASES-PRODUCING CARBAPENEM-RESISTANT GRAM-NEGATIVE BACTERIA

**FIGURE 1:**
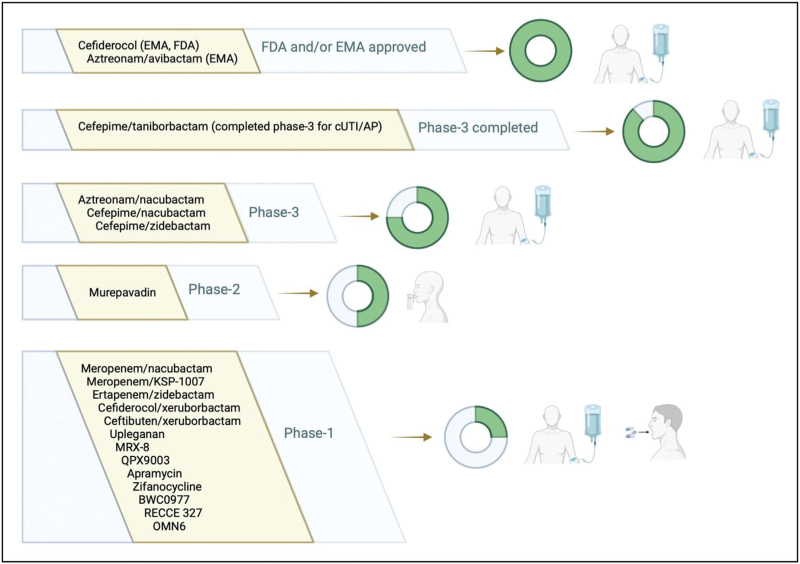
Recently approved antibiotics and antibiotics in different phases of clinical development with demonstrated or expected activity against MBL-producing CR-GNB. AP, acute pyelonephritis; CR-GNB, carbapenem-resistant Gram-negative bacteria; cUTI, complicated urinary tract infections; EMA, European Medicines Agency; FDA, Food and Drug Administration; MBL, metallo-β-lactamases. Green parts of circles represent the current degree of completeness of clinical development. To the right of the circles are the routes of administration (each route is represented if a related formulation is in development for at least one agent in a given phase of clinical development). Created with BioRender.com.

A graphical overview of currently approved antibiotics and antibiotics in different phases of clinical development with demonstrated or expected activity against MBL-producing CR-GNB is available in Fig. 1. Among currently marketed novel β-lactams and BL/BLI combinations, only cefiderocol shows activity against most MBL-producing CR-GNB [58]. An additional possibility involving marketed β-lactams and BL/BLI is that of combining ceftazidime/avibactam with aztreonam, thereby obtaining an aztreonam/avibactam combination able to exert activity against MBL-producing CR-GNB in those cases where synergistic activity of the two agents is demonstrated *in vitro*[6,59]. Notably, aztreonam/avibactam as a ‘single’ agent has been recently approved by the EMA, and will consequently require standard susceptibility testing without the need for a more laborious synergy testing (as when combining ceftazidime/avibactam and aztreonam). Given the above consideration, it is thus reasonable to expect a forthcoming future where clinicians will have two agents (cefiderocol and aztreonam/avibactam) for approaching empirical and targeted treatment of severe MBL-producing CR-GNB infections. From this standpoint, it would be essential to understand (and exploit) even slightly differences between the two agents to optimize therapeutic algorithms and guarantee the best suitable treatment for any given patient (considerations to be weighed in the balance, at least for empirical therapy, should include an updated local microbiological epidemiology of both phenotypical and molecular resistance to these novel agents). Furthermore, if approved by regulatory agencies in case of favorable results from ongoing (and, for some agents, already completed) phase-3 studies, also other novel agents (cefepime/taniborbactam, cefepime/zidebactam, cefepime/nacubactam, and aztreonam/nacubactam) could become available in clinical practice in the next few years and would require careful assessment for optimal inclusion within therapeutic algorithms for the treatment of severe infections by MBL-producing CR-GNB. Although necessary, this optimization could prove to be a difficult task. Indeed, as discussed in the previous section of this manuscript, approval of current and (possibly of) future agents in phase 3 of clinical development with demonstrated in vitro activity against MBL producers is and will be based on RCTs only including a very few patients with infections caused by MBL producers. From this standpoint, it could be essential to rapidly conduct large and well designed multinational observational studies, with proper adjustment for confounding, to understand the real world efficacy and safety of these novel agents in the treatment of MBL-producing CR-GNB infections and perfectionate their positioning within dedicated therapeutic algorithms. In turn, this is essential to guarantee both proper use for the single patient (maximization of efficacy in patients with MBL-producing CR-GNB infections) and preservation of activity of novel agents in the long term, avoiding selective pressure for resistance whenever their use is not necessary according to antibiotic stewardship core concepts. Against this backdrop, the increasing use of rapid molecular tests able to rapidly detect the presence of MBLs within clinical specimens could be (and partly, already is) crucial in helping both to rapidly deescalate to other agents (when MBL enzymes are not detected) or to more rapidly initiate agents active against MBL producers (when MBL enzymes are unexpectedly detected). Nonetheless, considering the possible development and diffusion of phenotypical resistance to novel agents also in presence of MBLs, updated local microbiological epidemiology data (to know *a priori* whether resistance to novel agents is possible even in presence of expected anti-MBL activity) should be coupled with use of rapid molecular tests for detection of MBLs. Looking at the future, the possible diffusion of reliable rapid phenotypical antibiotic susceptibility tests could further change this scenario, and consequently the approach to rapid diagnosis and treatment of MBL-producing CR-GNB infections.

Finally, regarding novel agents under clinical development and belonging to antibiotic classes other than β-lactams and BL/BLI, interesting agents are being developed exploiting both classical and novel mechanisms of action. However, as discussed in previous sections, almost all of them are still in phase-1 of clinical development. Therefore, they currently seem still far from possibly reaching clinical practice, with β-lactams and BL/BLI remaining the most reasonable classes of novel compounds expected to improve our armamentarium against MBL-producing CR-GNB in the next few years.

## CONCLUSION

After several years of polymyxin-based treatments as first-line treatment option, the availability of a few already approved β-lactams and BL/BLI combinations and of many other BL/BLI in late phases of clinical development showing activity against MBL-producing CR-GNB is revolutionizing the treatment of severe infections caused by these organisms. Improving the use of these agents through virtuous antimicrobial stewardship frameworks able to guarantee both the efficacious treatment of infections requiring their use and the avoidance of their use whenever not necessary (e.g., by not adopting de-escalation to other agents when infections by MBL-producing CR-GNB is excluded) remains a challenge of utmost importance that should not be overlooked.

## Acknowledgements

*None.*

### Financial support and sponsorship

*None.*

### Conflicts of interest

*Outside the submitted work, Matteo Bassetti has received funding for scientific advisory boards, travel, and speaker honoraria from Cidara, Gilead, Menarini, MSD, Mundipharma, Pfizer, Shionogi. Outside the submitted work, Daniele Roberto Giacobbe reports investigator-initiated grants from Pfizer, Shionogi, BioMérieux and Gilead Italia, and speaker/advisor fees from Pfizer, Menarini, and Tillotts Pharma. The other authors have no conflicts of interest to disclose.*

## REFERENCES AND RECOMMENDED READING

Papers of particular interest, published within the annual period of review, have been highlighted as:

▪ of special interest
▪▪ of outstanding interest
